# Stabilization of Charge Density Waves in Atomic Chains on Xenes

**DOI:** 10.3390/ma18163843

**Published:** 2025-08-15

**Authors:** Tomasz Kwapiński, Marcin Kurzyna, Mariusz Krawiec

**Affiliations:** 1 Institute of Physics, Maria Curie-Sklodowska University, 20-031 Lublin, Poland; 2 Institute of Computer Science and Mathematics, Maria Curie-Sklodowska University, 20-031 Lublin, Poland; marcin.kurzyna@mail.umcs.pl

**Keywords:** atomic chains, 2D surfaces, density of states, charge waves

## Abstract

We investigate the electronic properties of atomic chains placed on group-14 two-dimensional materials, Xenes, by analyzing the local electronic properties. Our results show that the hybridization between the chain and the substrate leads to significant modifications in the local density of states at each chain site, including peak splitting, broadening, and asymmetry. These effects are particularly pronounced for plumbene. Owing to the substrate’s V-shaped-like density of states, the chains exhibit strong localization effects and significant intensity variations in the electronic energy spectrum. In addition the present analysis reveals the emergence of charge density waves in atomic chains, for which the appearance and stability conditions are identified and provided. The charge density waves are more pronounced and stabilized by a specific electronic spectrum of Xenes, allowing them to penetrate deeper into the chain interior. Our findings contribute to the broader understanding of the interaction between one-dimensional chains and two-dimensional Xene materials, which have significant implications for developing advanced hybrid nanostructures and next generation electronic devices.

## 1. Introduction

One-dimensional (1D) systems such as atomic chains are fundamental building blocks of nanoelectronics, as these chains represent the thinnest possible conductors of electric current. Many intriguing physical phenomena have been observed in these systems, including Luttinger liquid behavior, charge waves, and electron and spin pumps [1,2,3], as well as more exotic effects like Majorana topological states [4,5], Floquet topological insulators [6], and time crystals [7]. These 1D systems, in the form of arrays of quantum dots (where all system parameters can be controlled using auxiliary electrodes) or atomic chains, are in direct contact with their environment, which is typically a substrate. Thus the wave functions of these structures hybridize with the electronic states of the substrate, leading to a renormalization of the molecular states. Therefore, understanding the electronic properties of such chains on different substrates is crucial for nanoelectronics.

Atomic chains can be experimentally fabricated through the self-assembly of atoms on crystalline surfaces, which means the chain is placed on a substrate. There are many experimental examples of such atomic chains at various vicinal and flat surfaces, including Si(113)-Pb [8], Si(335)-Au [9], Si(557)-Au [10,11], or Si(553) [12]. It is important to note that the silicon surface on which atomic chains are fabricated is usually stabilized by gold atoms. These gold atoms form a double chain embedded between silicon atoms on each terrace [13]. Such a terrace can be considered an effective two-dimensional (2D) surface, which is conductive, in contrast to the atoms in the bulk of the sample, which exhibit typical three-dimensional (3D) semiconductor properties. As a result, atomic chains formed on this surface are primarily coupled to the 2D structure that constitutes the outermost layer of the substrate.

Recently, other 2D systems with remarkable electronic properties have attracted much attention. This new class of 2D systems, known as 2D-Xenes, features single layers of atoms arranged in a honeycomb lattice [14,15,16,17,18]. The precursor of these materials is graphene, isolated in 2004 [19]. It consists of carbon atoms with $sp^{2}$-hybridized electronic orbitals giving rise to linear bands in the electronic spectrum, characteristic of Dirac particles. These bands originate from the π overlap of the $p_{z}$ orbitals. Other group-14 Xenes, like silicene, germanene, stanene, and plumbene, also exhibit the Dirac physics, even though their atomic structure is not completely planar, like in graphene, and the mixed $sp^{2}/sp^{3}$ hybridization appears. As a result, the π bands change their orbital character, and become substantially narrower than in the case of graphene. Owing to their remarkable electronic properties, 2D Xenes can possibly serve as templates for atomic chains, controlling and tuning their properties.

Charge density waves (CDWs) in 1D can emerge in ordered systems where the potential is disturbed by imperfections such as lattice defects, impurities, or dislocations, as well as in confined atomic systems due to boundary effects [20,21,22,23,24]. This phenomenon was first predicted by J. Friedel and is characterized by oscillations in the electron density around impurity sites [25], and it was initially observed using scanning tunneling microscopy on copper surfaces [26], where standing wave patterns were detected near atomic steps and point defects. The decay rate of these oscillations exhibits sinusoidal oscillations that decrease with distance from the perturbation and is strongly influenced by the dimensionality of the system [20] and can affect both the stability and physical properties of metallic atomic chains and ultrathin metal films [27]. It was also shown that both the conductance oscillations and the localized charge along the chain fade away due to the influence of the simple (structureless) substrate electrode [28].

CDW have gained increasing attention for their potential in future nanoscale and quantum technologies. Their tunable nature, sensitivity to external stimuli (such as electric fields, strain, or doping), and ability to exhibit non-linear electrical transport make them promising for applications in ultrafast switches [29], non-volatile memory devices, and neuromorphic computing architectures [30,31]. Moreover, their coherence and wave-like behavior open up possibilities in coherent signal processing and quantum information platforms [32]. These unique properties are especially advantageous when integrated with 2D materials and low-dimensional structures, offering compatibility with emerging low-power, high-density device paradigms. Studying 1D systems, such as atomic chains placed on 2D materials, is important both from a fundamental and an applied perspective. One-dimensional systems exhibit strong electronic localization, and collective phenomena like CDWs. When coupled to 2D substrates, such as Xenes, analogues of graphene, their electronic properties can be tuned via hybridization with the substrate’s electronic structure. These 1D/2D hybrid systems offer a unique platform for engineering novel quantum states and designing functional elements for molecular electronics, spintronics, and nanoelectronics. Therefore, understanding and controlling these hybrid structures is a promising direction for the development of future nanoscale and quantum technologies.

It is therefore essential to undertake studies that determine whether CDW can exist in realistic 1D systems, where the atomic chain is coupled to a substrate electrode with a specific and complex density of states (DOS). The commonly used wide-band limit (WBL) approximation for electrodes is merely a simplified toy model, which may be valid for 3D metallic materials but represents an oversimplification when applied to 2D atomic surfaces. In such cases, it fails to accurately capture the true physical behavior. In particular, it is inadequate for describing materials with a linear dispersion in their spectral function, such as in the case of Xenes. To the best of our knowledge, there are no theoretical works that resolve the question of whether CDW can exist in 1D systems coupled to realistic 2D surfaces, which should be thoroughly explained.

The primary objective of our work is to analyze the electrical properties of deposited atoms or atomic chains placed on 2D Xenes characterized by a linear energy dispersion around the Fermi level—such as graphene, silicene, germanene, stanene, and plumbene—and to compare these results with those for a metallic substrate having a flat DOS. We anticipate that the heterogeneous DOS of 2D materials, with numerous van Hove singularities, will be reflected in the local DOS structure on the atoms within the chain and can influence its electrical properties and charge distribution along the system. In this context, we investigate whether CDW can develop along the chain on 2D templates. While such waves are known to occur for chains on flat surfaces described by the wide-band limit approximation [23,28], they may be significantly suppressed or might not emerge at all on more complex substrates.

For a regular 3D crystal, the energy-band dispersion can be obtained analytically within the simple tight-binding approach. Similarly, for some 2D lattices (e.g., square, honeycomb, Lieb, and Kagome), the energy dispersion can be expressed in terms of elliptic integrals, featuring a van Hove logarithmic singularity in the band [33,34]. However, in our calculations, we focus on realistic description of the electronic band structure with aid of the density functional theory (DFT). In this way, we are able to handle details of the electronic structure of real Xenes in a wide range of energies and consider them as concrete templates to host adsorbed atomic chains. In the present studies, by utilizing the computed substrate DOS and applying the second quantization method together with the equation of motion for the retarded Greens function, we determine the local DOS for each atom in the chain deposited on the given Xene. Knowledge of the local DOS enables us to compute the charge occupancies on each atom and to analyze the charge density waves along the chain.

## 2. Theoretical Description

### 2.1. DFT Calculations

First-principles density functional theory calculations were performed within Perdew–Burke–Ernzerhof (PBE) [35] generalized gradient approximation to the exchange–correlation interaction as implemented in VASP 6.4.2 (Vienna ab initio simulation package) [36,37]. The core electrons were treated within the projector-augmented wave method [38]. The kinetic energy cutoff of 550 eV for the plane wave expansion of single particle wave function was used. The convergence criterion of total energy was chosen to be 10^−7^ eV. The Brillouin zone was sampled by a 24×24×1 Monkhorst–Pack k-points grid, including the Γ point [39] during the geometry optimization and 144×144×1 in the calculations of the density of states. The Xene atomic structure was modeled by a single atomic layer separated by 10 Å wide vacuum gap. The atomic positions were relaxed by a conjugate gradient method until the largest force in any direction was below 0.001 eV/Å.

**Figure 1:**
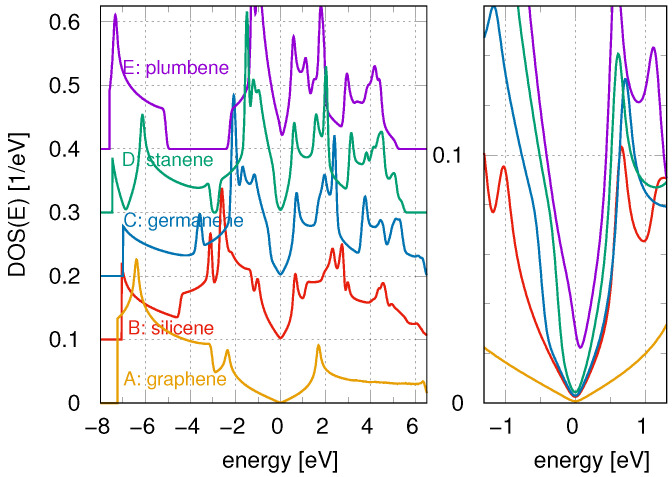
DFT calculations of the surface DOS for various 2D structures—graphene, silicene, germanene, stanene and plumbene (labeled A to E). For clarity, in the left panel, curves B through E have been shifted upward by 0.1, 0.2, 0.3 and 0.4, respectively.

Figure 1 presents the substrate DOS for various 2D Xenes—graphene, silicene, germanene, stanene, and plumbene—calculated using DFT, shown over a wide energy range (left panel) and a narrow range around the Fermi energy (right panel). In the left panel, curves B–E are vertically shifted for clarity. As can be seen, each DOS extends over an energy range of approximately ±8 eV and exhibits multiple peaks resembling van Hove singularities observed in ideal square or hexagonal lattices. They come from weakly dispersive and flat electron bands of post-graphene Xenes and weaker π-π overlap due to the larger atomic radius of Si, Ge, Sn, and Pb atoms (see Figure 2 of Ref. [18] and discussion therein). The DOS profile for all Xenes, except plumbene, is characterized by an almost zero value at the Fermi level ($E_{F}$) and a linear dispersion forming a V-shaped structure. In the case of plumbene, however, this behavior is disrupted by σ bands crossing the Fermi level, resulting in a metallic character [18] and a finite DOS at $E_{F}$ (right panel). It is also worth noting that curves B–E, corresponding to silicene through plumbene, exhibit qualitatively similar DOS features, in contrast to graphene, which displays a distinct DOS structure characterized by a broad and smooth V-shaped profile at the Fermi level and a relatively small number of van Hove singularities.

### 2.2. TB Calculations

To study the electronic properties of atomic chains on various Xenes, we employ the tight-binding technique, modeling the system as a substrate with a given DOS with an attached atomic chain. The chain of length *N* is composed of linear atomic sites which are coupled together by hybridization matrix elements *t* and characterized by on-site electron energies $\varepsilon_{i}$. The model Hamiltonian in the standard second-quantized form can be expressed as a sum of the following terms:

(1) $$\begin{array}{ccc} H & = & \sum_{i=1}^{N}\varepsilon_{i}a_{i}^{†}a_{i}+\sum_{\vec{k}}\varepsilon_{\vec{k}}a_{\vec{k}}^{†}a_{\vec{k}}+\sum_{i=1}^{N-1}t_{i,i+1}a_{i}^{†}a_{i+1}+\sum_{i=1}^{N}\sum_{\vec{k}}V_{i,\vec{k}}a_{\vec{k}}^{†}a_{i}+H.c. \end{array}$$

In the above equation, the first term represents the on-site electron energy within the chain, while the second term corresponds to the energy of the surface electrons, $\varepsilon_{\vec{k}}$, characterized by wave vectors $\vec{k}$. The operators $a_{i}$ ($a_{i}^{†}$) annihilate (create) an electron at the *i*-th site of the chain (i=1,…,N), and $a_{\vec{k}}$ ($a_{\vec{k}}^{†}$) are the corresponding annihilation (creation) operators for the leads. The third part of the Hamiltonian describes electron transitions along the chain (between atomic sites), and between the substrate and chain sites, respectively. Here, the spin index is omitted, as we are not interested in the magnetization effects, and both spin directions are considered independent.

Charge waves along the chain require an analysis of charge occupations at each site of the chain, $n_{i}$. These on-site occupations can be determined based on the relation

(2) $$\begin{array}{c} n_{i}=\int_{-\infty }^{E_{F}}LDOS_{i}\left(E\right)dE\;, \end{array}$$

where $LDOS_{i}\left(E\right)$ is the local DOS function at a given *i*-th site and can be obtained from the corresponding diagonal matrix elements of the retarded Green’s function $LDOS_{i}\left(E\right)=-\frac{1}{\pi }ImG_{ii}^{r}\left(E\right)$. This function can be derived using the equation of motion technique [40,41] from the relation $EG_{ij}^{r}\left(E\right)=\langle {\left[a_{i},a_{j}^{†}\right]}_{+}\rangle +{\langle \langle {\left[a_{i},H\right]}_{-};a_{j}^{†}\rangle \rangle }_{E}$. It allows us to express the following matrix relation: $\hat{A}\;\cdot {\hat{G}}^{r}=\hat{I}$, where $\hat{I}$ is the unit matrix, $\hat{G^{r}}$ represents a square N×N matrix of $G_{ij}^{r}\left(E\right)$ functions, and $\hat{A}$ is a complex matrix with elements

(3) $$\begin{array}{ccc} A_{i,j}\left(E\right) & = & \left(E-\varepsilon_{i}\right)\delta_{i,j}-t_{i,j+1}\left(\delta_{i,j+1}+\delta_{i+1,j}\right)-\Sigma_{i,j}\left(E\right). \end{array}$$

Here, $\Sigma_{i,j}\left(E\right)=\sum_{\vec{k}}V_{i,\vec{k}}^{*}V_{j,\vec{k}}{\left(E^{+}-\varepsilon_{\vec{k}}\right)}^{-1}$, and in general $V_{i,\vec{k}}^{*}$ electron hybridization elements between chain sites and the substrate Bloch states depend exponentially on the spatial positions of atoms [28,42]. Thus, the off-diagonal elements of $\hat{\Sigma }\left(E\right)$ rapidly decrease even for neighboring atoms and are therefore neglected in our calculations. All the information about the surface is contained in the diagonal elements of $\Sigma_{ii}\left(E\right)$, which can be expressed as $\Sigma_{ii}\left(E\right)=\Lambda_{ii}\left(E\right)-i\Gamma_{ii}\left(E\right)/2$, where $\Gamma_{ii}\left(E\right)=2\pi \sum_{\vec{k}}{\left|V_{i,\vec{k}}\right|}^{2}\delta \left(E-\varepsilon_{\vec{k}}\right)$, and for $|V_{i,\vec{k}}{|}^{2}$ independent of the wave vector $\vec{k}$, as well as for all atoms coupled to the same surface, we can write $\Gamma_{ii}\left(E\right)=2\pi {\left|V_{\vec{k}}\right|}^{2}DOS\left(E\right)$. The function DOS(E) stands for energy-dependent DOS of the Xene, which in our calculations is obtained from the DFT technique. The second function $\Lambda_{ii}\left(E\right)$ is related to $\Gamma_{ii}\left(E\right)$ through the Hilbert transform $\Lambda_{ii}\left(E\right)=\frac{1}{2\pi }\int_{-\infty }^{\infty }\Gamma_{ii}\left(E^{\prime }\right)/\left(E-E^{\prime }\right)dE^{\prime }\;.$ Thus, knowledge of the Xene DOS is sufficient to compute both functions $\Lambda_{ii}\left(E\right)$ and $\Gamma_{ii}\left(E\right)$, which depend on energy and, once substituted into Equation (3), allow for the calculation of the retarded Green’s function by inverting the $\hat{A}$ matrix:: $G_{ii}^{r}\left(\varepsilon \right)={\left({\hat{A}}^{-1}\right)}_{ii}=\mathrm{cof}{\hat{A}}_{ii}/\det\hat{A}$, where $\mathrm{cof}({\hat{A}}_{ii})$ denotes the algebraic complement of ${\hat{A}}_{ii}$, and $\det(\hat{A})$ represents the determinant of the matrix $\hat{A}$. Consequently, we can determine the local DOS and charge occupations at each site of the chain. It is worth noting that for specific forms of the substrate DOS (e.g., van Hove type, rectangular, or elliptical DOS), an analytical form of the Hilbert transform exists, which allows for explicit analytical expressions for the Green’s functions. Also for a regular chain on a substrate described by energy-independent DOS (wide-band approximation) with homogeneous on-site energies and uniform atom–atom couplings, the Green functions can be expressed analytically using Chebyshev polynomials of the second kind [43]. In this paper, due to the non-analytical substrate DOS of 2D structures, we obtain the local DOS along the chain and the electron occupancy numerically.

In our calculations, we employ the zero-temperature limit, and all energies are expressed in units of $\Gamma_{0}=1$ eV (where we use the relation $\Gamma_{0}=2\pi V_{\vec{k}}^{2}/w$ for a flat, $\vec{k}$-independent Xene DOS of width w=20). The energy reference point is set at the chemical potential of the lead, $E_{F}=0$. For parameters used in the paper, the typical coupling strengths between atomic sites range from 0.1 to 4 eV.

## 3. Results and Discussion

### 3.1. Few-Atom Systems

**Figure 2:**
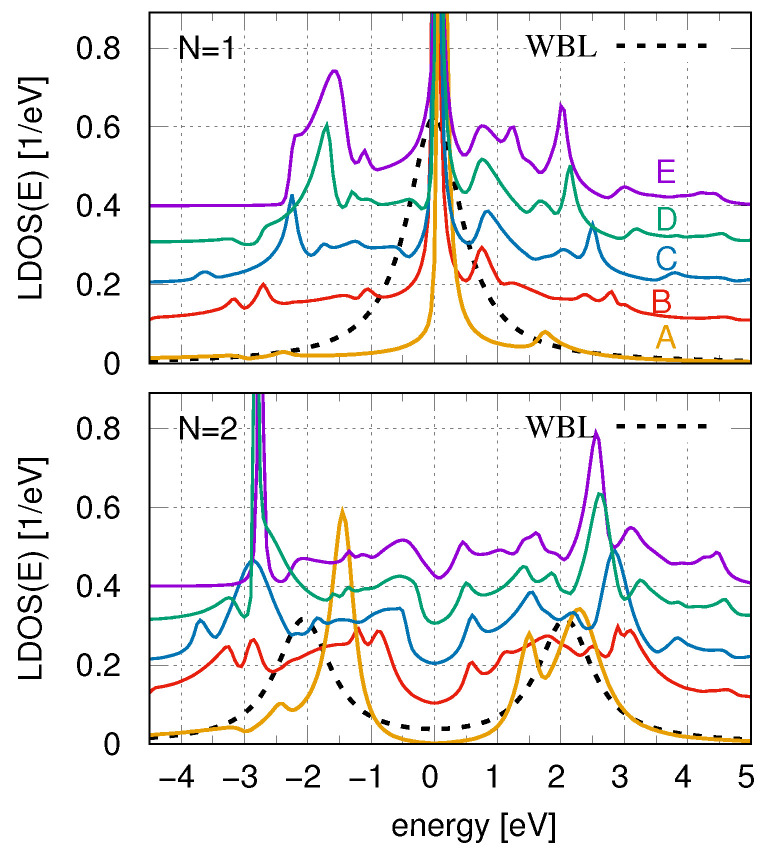
Local DOS for a single atom (upper panel, N=1) and for two coupled atoms (bottom panel, N=2) placed on different 2D surfaces. The corresponding surface DOS are shown in Figure 1 (the colors of the curves and letters A–E correspond to the surface DOS lines). The dashed curves represent the local DOS at the atoms for the surface DOS obtained within the wide band approximation (WBL). The other parameters are $\varepsilon_{i}=\varepsilon_{0}=0$, $t_{12}=2$, $\Gamma =1\Gamma_{0}$.

Before analyzing the electronic properties of atomic chains, we first examine how the local DOS is modified for a single atom and for an atomic dimer (two coupled atoms). The upper panel of Figure 2 presents the results for a single atom placed on various 2D Xenes, ranging from graphene to plumbene, represented by curves A–E, with the corresponding substrate DOS shown in Figure 1. Additionally, the figure includes a case where the atom is placed on a surface characterized by a flat, structureless DOS, modeled within the wide-band approximation, shown as a dashed curve.

For the latter case, the LDOS exhibits a smooth Lorentzian shape with a maximum at $E=\varepsilon_{0}=0$ (upper panel). However, for the 2D Xenes, the width of the main LDOS peak is significantly narrower, and its intensity is considerably higher compared to that obtained from calculations using a flat DOS surface. This is because the atomic single-particle energy level lies at the system’s Fermi level, where the substrate DOS of Xenes is very low. Consequently, the atomic state more closely resembles that of an isolated atom, characterized by high intensity and narrow broadening. Moreover, when the actual substrate DOS is taken into account, the atomic LDOS exhibits multiple smaller peaks and irregularities in addition to the main peak. These additional features closely reflect the structure of the Xenes DOS. For instance, in the case of plumbene, pronounced peaks appear in the substrate DOS at approximately E=−1.5, +0.6, +1.1, +1.9, +2.9, and +4.1, with corresponding peaks observed in the atomic LDOS at similar energies (curve E). It should be noted, however, that these additional LDOS peaks are slightly shifted along the energy axis due to the nonzero real part of the self-energy (in the Hilbert transform). As a result, the actual positions of the LDOS peaks for plumbene are located at E=−1.6, +0.7, +1.2, +2, +3, and +4.4, respectively. Nevertheless, the intensity of these additional peaks decreases with increasing distance from the main $\varepsilon_{0}$ peak, which explains why the LDOS peaks at E=+3 and +4.4 are only faintly visible. Note that the LDOS of a single atom on graphene—whose DOS is relatively smooth near the Fermi energy—more closely resembles that predicted by the wide-band limit approximation (black and yellow curves).

For a system of two coupled atoms on the surface, the situation is somewhat different (bottom panel of Figure 2). In the case of a flat WBL surface, the LDOS at both chain sites is identical and is characterized by two distinct peaks located at energies $E=\varepsilon_{0}\pm t_{12}=\pm 2$, with a local minimum at the Fermi level (black dashed line). When such an atomic dimer is placed on a substrate with a relatively smooth DOS, such as graphene, the LDOS exhibits a structure broadly similar to that of the WBL case, except that the peak at positive energies becomes noticeably split. This splitting arises because the graphene substrate has a local DOS maximum around E=+1.8, which is close to the molecular dimer peak at E=+2. As a result, both features are reflected in the LDOS curve (yellow line). This effect becomes even more pronounced for dimers placed on other 2D substrates, where the coexistence of molecular states and substrate states at similar energies leads to noticeable LDOS peak splitting. For instance, at positive energies, plumbene exhibits a strong substrate DOS peak at E=+1.9. The interaction between this substrate state and the molecular dimer state at E=+2 results in the LDOS exhibiting renormalized peaks at E=+1.5 and +2.5, with a local minimum at E=+2 (purple line in the bottom panel). A similar LDOS splitting is observed for other 2D substrates, including silicene, germanene, and stanene. A comparable effect also occurs at negative energies around the molecular peak at E=−2. Moreover, for some substrates, significant changes in peak intensity are observed on LDOS curves, particularly around E=−2.7 (green and purple curves for stanene and plumbene, respectively). This behavior is attributed to the very low substrate DOS in this energy region, which leads to weak dispersion of the states located there. As a consequence, these states exhibit strongly enhanced intensity in the LDOS.

### 3.2. Atomic Chains on Various 2D Substrates

Quantum systems composed of groups of atoms are characterized by the presence of molecular states, whose number usually equals the number of constituent atomic states. These molecular states hybridize with the substrate, resulting in each atom being described by a local DOS that typically contains as many peaks as there are molecular states, with varying intensities. Similarly, linear arrays of atoms forming atomic chains on a surface exhibit analogous behavior. In this section, we analyze the electronic properties of such chains on various substrates and investigate the possibility of charge density waves forming within these systems.

**Figure 3:**
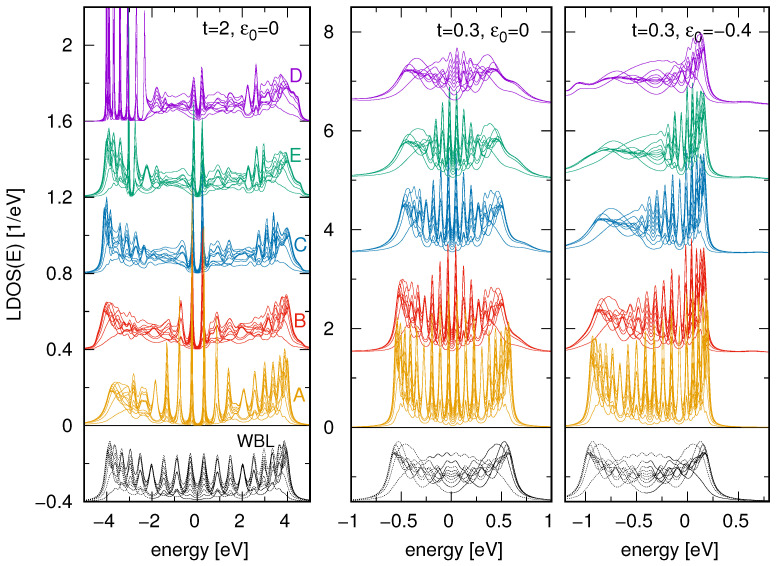
Local DOS at each atomic site of a chain of length N=20 located on various 2D surfaces as described by the DOS shown in Figure 1 (the colors of the curves and the letters A–E correspond to the surface DOS lines). The bottom black curves represent the local DOS at the atoms for the surface’s WBL DOS. The left panel corresponds to strongly coupled atomic sites, t=2, and $\varepsilon_{0}=0$, Γ=0.25. The middle and the right panels show the results for weakly coupled sites, t=0.3, and for $\varepsilon_{0}=0$ and $\varepsilon_{0}=-0.4$, respectively, and for Γ=0.1. All curves (besides A lines) are shifted for better visualization.

*Strongly coupled sites*. At the outset, we consider an atomic system on a 2D surface consisting of N=20 closely spaced sites, which leads to large overlap integrals of the wave functions and, consequently, significant coupling between neighboring atoms, denoted by *t*. This implies that the energy bandwidth of such a chain extends over approximately ±2t, thereby covering a substantial portion of the substrate’s DOS. Selected calculations of the local DOS for this chain (computed at each atomic site) are presented in the left panel of Figure 3. As before, various substrates have been considered—from a flat surface described within the wide-band limit (represented by the black curves in the bottom), through graphene (yellow curves, labeled A) to plumbene (violet lines, labeled E). On the WBL substrate, the chain exhibits 20 LDOS peaks with comparable dispersion and intensities (each black curve representing the LDOS at a different atomic site). All these curves are symmetric with respect to the Fermi energy, $E_{F}=0$, and display spatial symmetry (i.e., the LDOS at sites 1, 2, … is identical to that at sites N,N−1,…, respectively). In contrast, for the 2D substrates, the LDOS functions exhibit distinct characteristics. Notably, the chain placed on graphene shows very high and narrow LDOS peaks near the middle of the band, which is associated with the very low substrate DOS in the vicinity of the Fermi energy—a feature that is also present, albeit to a lesser extent, in the remaining substrates (B–E), all of which exhibit a DOS minimum at $E_{F}$. Moreover, some curves display pronounced energy asymmetry, particularly for the plumbene and stanene substrates (curves D and E). This asymmetry originates from the intrinsic asymmetry of the surface DOS in these materials, combined with the notably low DOS values in the negative energy region around E=−4 (see Figure 1).

*Weakly coupled sites.* In the case of chains with weakly coupled atoms (i.e., with a small inter-site hopping integral), the bandwidth of the chain’s DOS is narrow and may encompass only a small portion of the substrate’s DOS. The region around the Fermi energy is particularly interesting, as the substrate DOS exhibits a linear dispersion there as shown in Figure 1. The LDOS functions of such weakly coupled chain at different substrates are shown in Figure 3, middle and right panels, for every chain site. Note that when the single-particle energy levels of the chain are precisely aligned with the Fermi level ($\varepsilon_{0}=0$, middle panel), all LDOS curves are symmetric with respect to the Fermi energy since the substrate DOS functions are also symmetric in that region (compare with the curves in the right panel of Figure 1). It is also worth noting the variation in the intensity of the LDOS peaks, from the highest for the graphene substrate (yellow lines) to the lowest for plumbene (violet lines). This effect is closely related to the substrate DOS values near the Fermi energy—the lower the DOS value, the more the electronic states resemble isolated atomic states (i.e., with narrow dispersion and high intensity). Furthermore, for substrates with a very low DOS around the Fermi level, the molecular states visible in the chain LDOS are well separated (small dispersion), whereas, for example, on a WBL substrate (black lines) or on plumbene (violet lines), the LDOS curves display only small local peaks, and due to the overlapping of these states, not all are clearly revealed.

In the right panel of Figure 3, we also analyze the case where the single-particle energies of the chain are slightly shifted relative to the system’s Fermi energy, setting $\varepsilon_{i}=\varepsilon_{0}=-0.4$. In this scenario, the LDOS curves for the chain on a surface with a structureless DOS (modeled within the WBL) are identical to those shown in the middle panel, except for a uniform shift along the energy axis by −0.4. In contrast, significant differences are observed for the 2D Xene substrates. Notably, the LDOS curves for each Xene exhibit a pronounced asymmetry—the high-intensity peaks on the right-hand side of the plots result from the low substrate DOS in that energy region (near the Fermi energy) as discussed earlier. Conversely, in the energy range around E≃−1, the substrate DOS is considerably higher, which leads to a broadening and partial overlap of the LDOS peaks for the chain. As a result, the LDOS in that region (the left side of the plots) appears relatively smooth. The most pronounced differences in the LDOS curves occur between the graphene substrate (yellow lines) and the plumbene substrate (violet lines), reflecting the respective DOS profiles of these substrates in the considered energy range.

**Figure 4:**
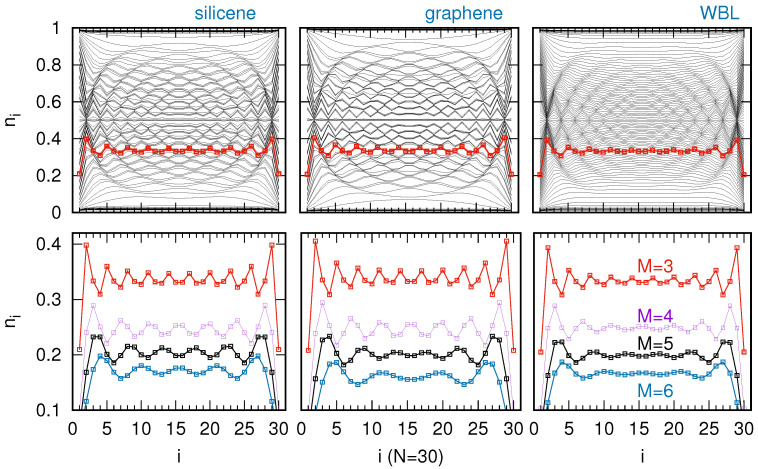
Charge occupancies at every chain site *i* for the chain length N=30, for various 2D surfaces: silicene, graphene, and flat WBL (from left to right panels). The upper panels show $n_{i}$ curves for different chain on-site energies from $\varepsilon_{0}=-4$ (upper curves) up to $\varepsilon_{0}=4$ (bottom curves) and the red-color curves represent the case for $\varepsilon_{0}=3.24$. The bottom panels show occupancy curves for special values of $\varepsilon_{0}$, which correspond to the specific oscillation period M=3, $\varepsilon_{i}=t=2$, M=4, $\varepsilon_{0}=\sqrt{2}t=2.83$, M=5, $\varepsilon_{0}=\left(\sqrt{5}+1\right)/2t=3.24$ and M=6, $\varepsilon_{0}=\sqrt{3}t=3.46$, as is indicated in the right bottom panel. The other parameters are t=2, $\Gamma =0.25\Gamma_{0}$.

*Charge waves in atomic chains*. Knowledge of the local DOS enables the calculation of charge occupancies (Equation (2)) and analysis of charge density wave distributions along the chain. Figure 4 presents these CDWs along a chain of N=30 sites for exemplary silicene, graphene, and flat DOS substrates, shown in the left, middle, and right panels, respectively. The upper charge curves (black lines at the top of the panels) correspond to the single-particle energy level in the chain set to $\varepsilon_{0}=-4$, while the subsequent curves represent incremental increases in energy, culminating at $\varepsilon_{0}=+4$ for the lowest black curves. Analyzing these plots reveals a distinct pattern formed by the individual charge curves, characterized by specific concentrations (seen as darker areas) that indicate the emergence of charge waves in such systems. For instance, the red curves (highlighted for $\varepsilon_{0}=3.24$) display characteristic Friedel oscillations, with maximal amplitude at the chain edges that gradually decays toward the center. The charge distribution exhibits pronounced left–right symmetry with respect to the chain center, as well as particle–hole-like symmetry relative to $n_{i}=0.5$. Consequently, charge oscillations with identical periodicity emerge for both positive and negative $\varepsilon_{0}$.

Charge waves are characteristic of a regular chain on the WBL surface, for which we have a clear understanding of when such waves occur and their oscillation period as given by equation $E_{F}-\varepsilon_{0}=2t\cos\frac{l\pi }{M}$, where *M* is the oscillating period and l=1,...M−1, [23]. For irregular 2D surfaces, a more detailed analysis of the charge occupancies is required, which we perform in the lower panels of Figure 4. There, charge distributions with oscillation periods of 3, 4, 5, and 6 atoms for the chain on a WBL surface (right panel) are plotted (the specific energy values were obtained analytically from the above equation). For the same single-particle energy positions, charge waves are calculated for the chain on silicene and graphene surfaces (lower panels, left and middle parts, respectively). As can be seen, the amplitude of the charge oscillations for the 2D Xenes is significantly larger than for the WBL surface—on the WBL surface, these waves nearly vanish in the middle of the chain (for example, the blue line for the oscillation period of 6 atoms), whereas on the graphene (and other 2D Xenes) they remain clearly visible even at the center of the chain. This outcome is a consequence of the substrates: the WBL surface exhibits a flat DOS across the entire energy range, whereas the 2D Xenes feature a minimum DOS around the Fermi energy, which strongly enhances the LDOS peaks on the chain atoms (all DOS curves are normalized). Since charge oscillations require significant LDOS modulation at the Fermi level, the V-shaped DOS of the substrate induces charge oscillations along the chain with greater amplitude, allowing the wave to propagate much further along the chain.

It is important to note that, despite the complex DOS structure of the considered substrates, the oscillation period of the charge waves typically remains constant along the chain, with only a slight increase in oscillation amplitude observed on 2D substrates. Consequently, the results obtained for other 2D surfaces are qualitatively similar to those for silicene or graphene, with only quantitative differences arising from variations in the DOS values of the respective substrates. This behavior significantly simplifies the experimental observation of charge waves in chains placed on 2D materials compared to those on metallic surfaces modeled by WBL. However, for longer oscillation periods, the required single-particle energy levels are relatively far from the Fermi energy. For instance, in the case of a period of M=6, the corresponding energy level is $\varepsilon_{0}=3.46$. In such cases, the detailed structure of the 2D Xenes DOS at $E=\varepsilon_{0}$ starts to play a significant role in determining the site occupations. This may lead to distortions of the charge wave oscillation period along the chain. Such behavior is observed, for example, on graphene (as well as on plumbene and stanene—not shown here), where a 30-atom chain exhibits four distinct charge maxima, whereas on silicene, germanene, and the WBL surface, five clearly visible maxima are present, indicating charge oscillations with a perfect period of six atoms.

Finally, we would like to comment on possible experimental realization of the present findings, which might be a bit challenging, as, in principle, there are no reports on the synthesis of true atomic chains on Xenes. The oldest member of the family, graphene, faces the problem of weak interaction with adsorbates, which limits the most common ways of growing based on the self-assembly processes. On the other hand, the post-carbon Xenes have been grown only on very few substrates and their synthesis and functionalization still is in infancy. Nevertheless, some attempts could be made utilizing stepped surfaces. The most known vicinal Si surfaces already host one-dimensional chains, either their step edges or decorated metal atom chains, for example, on the Si(553)-Au surface, where fluctuating CDW states have been observed, and fractional solitons have appeared at the step edge [2,44]. For this surface, the bias-dependent fractional periodicity of the STM height profiles of Si atomic chains—which is closely related to the charge waves along the chain—has been reported in our previous paper [45]. This means that charge wave phenomena in 1D systems on vicinal surfaces are experimentally observable, and our work indicates that in the presence of a 2D Xenes layer, such oscillations can be significantly enhanced. The Si(553)-Au surface, in its native or atom-decorated form, with arrays of one-dimensional chains, can then be covered by graphene [46,47] and forming hybrid 1D/2D systems. Finally, other vicinal substrates could also be used to grow anisotropic graphene [48] or other Xenes, on which 1D self-assembly should be possible. One particularly interesting problem is the investigation of whether such waves can exist in 1D topological systems, where due to the chiral symmetry, an energy gap exists in the system around the Fermi energy. While the realization of such systems at the atomic scale remains challenging, we have recently shown that a topological phase can emerge in a Si atomic chain on the Si(553)-Au surface modified with indium atoms [49].

## 4. Conclusions

In this work, we investigated the electronic properties of atomic chains on various 2D Xenes, with a particular focus on how the substrate’s DOS influences the chain’s electron occupancies. We demonstrated that when an atomic chain is placed on a substrate with a nearly featureless DOS as described by the wide-band approximation, its molecular states appear symmetrically distributed around the Fermi level in the local DOS. However, for real 2D materials such as graphene, silicene, germanene, stanene, and plumbene, the chain’s LDOS undergoes significant modifications due to hybridization with the substrate states. In particular, the presence of sharp peaks in the substrate DOS leads to additional effects in the chain’s local DOS, including peak splitting and asymmetric broadening. This effect is most pronounced for substrates with a highly structured DOS, such as plumbene. Since 2D materials exhibit a very low DOS near the Fermi energy along with a linear DOS dispersion, this results in pronounced localization effects and significant intensity variations in the chain’s local DOS. For strongly coupled atomic sites in the chain, the electronic states extend over a broader energy range, interacting with multiple regions of the substrate DOS, which enhances hybridization effects. These findings provide valuable insights into how substrate-induced modifications influence the electronic properties of atomic chains, leading for example to almost dispersionless bounding states in LDOS.

Furthermore, our analysis reveals the emergence of charge density waves in atomic chains placed on various 2D substrates. The spatial modulation of site occupancies along the chain observed in our results suggests that CDWs are stabilized on 2D Xenes, as the oscillation amplitudes increase significantly, allowing the charge waves to penetrate deeper into the chain interior. This effect is highly relevant for experimental studies of charge waves in one-dimensional systems supported on 2D materials.

## Data Availability

The original contributions presented in this study are included in the article. Further inquiries can be directed to the corresponding authors.
